# Expanding opportunities for mining bioactive chemistry from patents

**DOI:** 10.1016/j.ddtec.2014.12.001

**Published:** 2015-02-10

**Authors:** Christopher Southan

**Affiliations:** IUPHAR/BPS Database and Guide to PHARMACOLOGY Web Portal Group, Centre for Integrative Physiology, School of Biomedical Sciences, University of Edinburgh, Hugh Robson Building, Edinburgh EH8 9XD, UK^1^

## Abstract

Bioactive structures published in medicinal chemistry patents typically exceed those in papers by at least twofold and may precede them by several years. The Big-Bang of open automated extraction since 2012 has contributed to over 15 million patent-derived compounds in PubChem. While mapping between chemical structures, assay results and protein targets from patent documents is challenging, these relationships can be harvested using open tools and are beginning to be curated into databases.

## Graphical abstract

**Figure:**
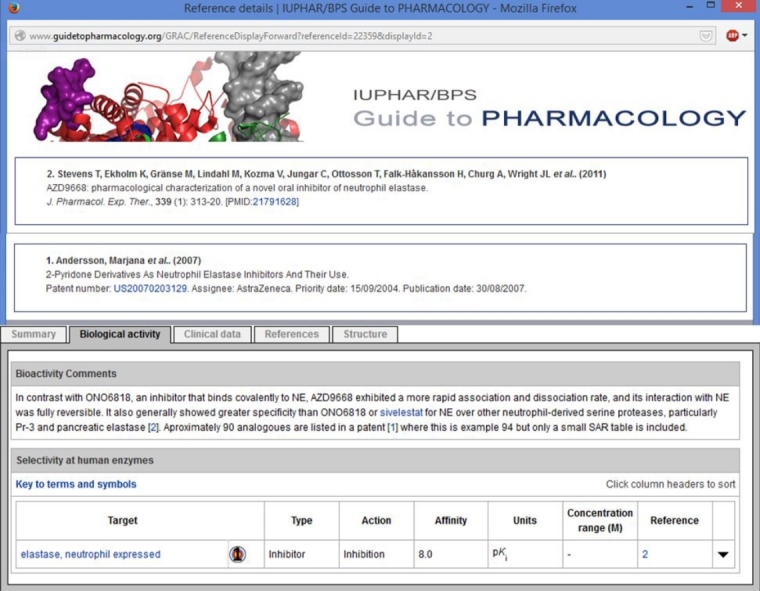

## Introduction

Compared to papers, patents in the biological sciences have hitherto been an underexploited information source, principally because retrieval specificity and data extraction is more challenging [1]. However, the World Intellectual Property Organisation (WIPO) indexing of 2.4 million PCT (WO) publications indicates 8.4% are assigned the International Patent Classification (IPC code) A61K (medical, veterinary science and hygiene) that encompasses bioscience filings [2]. Medicinal chemistry as C07D (heterocyclic compounds) represents 3.1%. This article focuses on the 1.5% subset of C07D (and) A61K filings for two main reasons. Firstly, the data mining challenges for bioscience patents as a whole are too diverse (including millions of sequence listings) to be covered here [3]. Secondly, for medicinal chemistry, patents have a central importance, because they not only underpin over four decades of drug discovery research (both commercial and academic) but also contain substantially more structure–activity relationship (SAR) results than journals. This article will focus on exploring scientific value rather than intellectual property (IP) because, while both aspects are intertwined, the analytical approaches diverge. A short video accompanies this article.

## Value

The question needs to be posed as to what data-centric patent mining has to offer practitioners in cheminformatics, pharmacology, medicinal chemistry or chemical biology. To answer this, it is necessary to compare the availability of data from non-patent sources. The appearance of ChEMBL in 2009 substantially increased the scale of results accessible from medicinal chemistry journals, with the current (release 19) count of 1.41 million structures including 0.94 million extracted from 57K papers (n.b. because ChEMBL subsume CIDs from confirmed PubChem BioAssays their compound count *in situ* exceeds the ChEMBL source count inside PubChem) [4]. However, specialised databases have been curating SAR data from the literature for some years prior to this, including BindingDB [5], Guide to PHARMACOLOGY (GtoPdb, formerly IUPHARdb) [6], GLIDA [7] and PDSP [8]. In addition, there are 0.41 million compounds flagged as ‘active’ in PubChem Bioassay (http://www.ncbi.nlm.nih.gov/pcassay) from sources other than ChEMBL.

**Figure 1:**
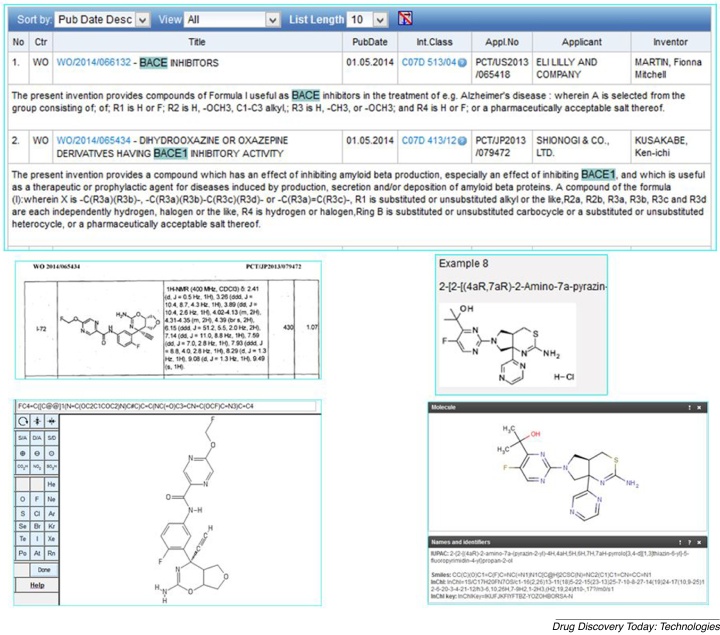
Finding and extracting selected examples from WO2014066132 and WO2014065434. In the upper panel the search term matches are highlighted in green. In the lower-left panel, example 72 from 5434 (page 238 in the PDF) was reported to have an IC50 against purified enzyme of 13.6 nM (page 249). The structure was determined from an initial image conversion using OSRA [11] and subsequently edited in the PubChem sketcher [12] from which PubChem searches were launched. The SMILES and InChIKey are shown below. FC4=C([C@@]1(N=C(OC2C1COC2)N)C#C)C=C(NC(=O)C3=CN=C(OCF)C=N3)C=C4 InChIKey = SOYARSISURDFSW-SKMDKRRUSA-N. In the lower-right panel example 8 from 6132 is shown (page 60 in the PDF) that has a reported IC50 of 105 nM (on page 63). Using chemicalize.org [13], the IUPAC name was used to generate a range of molecular outputs including a SMILES string and the InChIKey below. CC(C)(O)C1=C(F)C=NC(=N1)N1C[C@H]2CSC(N)=NC2(C1)C1=CN=CC=N1 InChIKey = IKIJFJKFIYFTBZ-YOZOHBORSA-N.

The utility of engaging with patents as an adjunct to non-patent sources can be introduced via a practical example. The WIPO database provides a searchable interface for patents from the major authorities. Executing a simple query (select for BACE* AND inhibitor(s), front page, English PCT applications) gives 280 results. The first two documents, both published on May 1st 2014, were WO2014066132 from Eli Lilley [9] and WO2014065434 from Shionogi [10], both specifying BACE1 inhibitors for Alzheimer's disease (6132 used BACE as a synonym for BACE1, UniProt P56817). These are shown in Fig. 1, together with the extraction of two example structures linked to activity data.

Connections gained from this result included the following:

1. Using the same search parameters, it was established that Shionogi had published nine BACE1 patents since 2007 and Eli Lilley five since 2005 (all potentially extractable as described in this section).
2. The rendering of images and tables in-line with text (except the Shionogi PDF exceeded the page limit) allowed scanning of results but full PDFs could be downloaded for checking.
3. The structure and associated IC50 values for selected potent BACE1 inhibitors, were discerned using chemicalize.org for the former and the result tables for the latter.
4. As ascertained by a PubChem search, example 8 from 6132 was identified in CID 73603937, deposited as the HCL by Thomson Pharma on 12th of May (presumably extracted from the same patent) but no close analogues were found.
5. While example 75 from 5434 had no exact match (i.e. was novel at that time) 60 analogues were found in PubChem by searching at 90% similarity. Many of these (e.g. CID 68164415) connected, via SureChEMBL, to a Roche BACE1/BACE2 filing, US20120202803 [14] and one (CID 71619629 via ChEMBL) connected between a Roche paper [15] and a Protein Data Bank experimental 3D ligand structure (4J0P).
6. In 6132, example 8 had cell line and mouse *in vivo* data (indicating this to be a possible lead compound).
7. 5434 describes 123 analogues with 15 specified sub-200 nM IC50s.
8. 6132 describes 114 analogues with three specified IC50s.
9. Detailed chemical synthesis, analytical data and assay descriptions in both documents.
10. 5434 cites 44 related patents and 3 papers, while 6132 cites one of each.
11. While chemicalize.org was able to automatically extract structures from the full text at Free Patents Online, example structures could only be extracted via their IUPAC name strings from 6132 (because structures in 5434 were image-only).
12. While medicinal chemistry papers from both companies have been extracted into ChEMBL (66 from Shionogi and 265 from Eli Lilly, including some on BACE1 inhibition) it could be at least 18 months before compounds from these patents appear in a journal article and sometime after that before a ChEMBL release surfaces the published structures in PubChem.
13. Since this search was carried out, both documents have been processed in SureChEMBL (n.b. this source is currently still designated SureChem for the pre-2013 entries in PubChem).

Aspects related to the search example are expanded as general points in Box 1. In addition, some of the themes are addressed in the following sections.

Box 1 **Utility and challenges of patent data**

Utility

• Relationships between the entities of document, assay description, assay result, compound structure and protein identifier (D-A-R-C-P) can be identified.
• Other target types (e.g. tumour cells, bacteria or protozoal parasites) may also have activity results.
• SAR tables (e.g. Ki or IC50) can include hundreds of novel structures.
• Only a proportion may appear in papers (roughly 10–30%) years later, or never.
• Many inventor teams include highly cited medicinal chemistry authors.
• Exemplified compounds are usually supported by synthesis descriptions and analytical data.
• The combination of references, plus the examiner's report, provides a citation network of papers and patents.
• The non-redundant medicinal chemistry corpus (as WO documents) is freely available for metadata querying and text mining from many open sources.
• Recent US patent XML includes electronic structures as Complex Work Units (CWUs), improved text quality and consequent better CNER extraction.
• Additional bioactivity data may be included (e.g. cell lines, rodent models, target specificity cross-screening, P450 profiling or other ADMET results).
• Patents may have complementary data content to journal publications, for example where chemotype similarity connections can be made via ChEMBL.
• Biological domain coverage can be widened (e.g. additional PCT codes for antinfectives, tropical diseases, pesticides and agrochemicals).
• The majority of exemplified patent chemistry is not only already in PubChem but also via the InChIKey, can be structure-matched by a Google search in about 0.2 s [21].

Challenges

• Some patents exemplify most, or even all, analogues without explicit activity results.
• Binned activity values are less useful than discrete ones for SAR modelling.
• Various forms of deliberate obfuscation are common, including uninformative titles, confusing or missing data relationships, virtual enumerations and prophetic protocols (i.e. not actually executed).
• Judging the scientific and technical quality of patent-only results is difficult.
• Complexity of large document sets related by patent family and kind codes, publishing identical content years apart.
• In terms of drug discovery, only a small proportion of C07D documents have SAR value, because a lead compound series first-filing is followed by many secondary patents from originators, competitors or generics companies.
• Open CNER resources extract all chemistry, regardless of IPC code. Thus, a proportion of structures (i.e. from patents not in C07D and A61K) have neither linked bioactivity nor biological relevance.
• Whereas patents quote relevant papers (as mandated for US applications), the citing of patents by journal authors is patchy.
• Even basic data content statistics are essentially unknown (e.g. the % documents using full IUPAC names vs images, or both, for exemplifications).
• Automatic extraction says nothing about associated IP claims. *Ipso facto* not all patent-extracted structures are ‘patented’ (but they are prior-art by definition).

## Speed

For operations filing patents that include novel compounds with commercially useful bioactivity, rapid interrogation of patent chemistry is an imperative. Because they are also likely to licence commercial databases for prior-art checking and competitive intelligence, the timing at which patent structures surface ‘in the wild’ is less of an issue (except to note that public sources must now be included in prior-art searches). Nevertheless, the scientific preview opportunities offered by medicinal chemistry patents can also be valuable for those not necessarily committed to filing themselves (Box 1). In this respect, it may not be appreciated how level the information playing field is become, because a patent becomes open and globally accessible only on the day of publication. This means that, as shown in Fig. 1, using relatively straightforward open resources and tools, key SAR can be extracted from the document within hours. In addition to day-one publication at the major offices, the lag-time for new patents to appear in open searchable sources such as Google Patents (http://www.google.com/advanced_patent_search), Free Patents Online (http://www.freepatentsonline.com/), Patent Lens (http://www.lens.org/lens/) and others, is now reduced to days. However (as specified in Table 1) current PubChem sources (except Thomson Pharma) have a submission lag for patent chemistry. However the recently enhanced SureChEMBL pipeline *in situ* (i.e. not yet in PubChem) now has a chemistry extraction time of less than a week. A practical preview example can be given for the case of BACE2 as a new diabetes target [16]. Only one targeted inhibitor has appeared in a 2011 paper but, since 2010, many hundreds have been exemplified as different chemotypes in patents published from four pharmaceutical companies and one academic institution. In addition, most of these included discrete activity data and comparative BACE1 cross-screening results. Thus, in the five years since the first published patents, no papers describing extensive chemistry directed against this important new therapeutic target have yet appeared.

**Table 1:** Comparative assessment of patent structures inside PubChem. The specified sources can be retrieved from PubChem by simple selects (e.g. ‘SureChem’[SourceName] but note this is now SureChEMBL) with the results as CID counts. PubChem and ChEMBL are included for comparison. Totals are in millions for each source and dates are from the last update. Subsequent columns are filters expressed as %. In order these are; stereo and E/Z (completely or partially unspecified), Mw < 400, unique structures (to that source), entries with two components, rule-of-five with 200-800 Mw range. Int. refers to links to patent documents provided inside PubChem. Ext. refers to document mappings in the source links

**Source**	**Total**	**Date**	**Stereo & E/Z**	**Mw** **<** **400**	**Unique**	**Mix.**	**ROF 2-800**	**Int. link**	**Ext. link**
**PubChem**	49.8	May-14	35%	58%	52%	2.5%	72%		
**IBM**	2.3	Jun-12	41%	71%	31%	0.3%	58%	Yes	Yes
**SureChem**	9.3	Mar-13	38%	52%	52%	5.9%	63%	No	Yes
**SCRIPDB**	3.9	Aug-12	29%	48%	27%	5.4%	56%	Yes	Yes
**ChEMBL**	0.9	Apr-14	26%	48%	17%	1.9%	61%		
**Thomson Pharma**	4.2	May-14	25%	49%	15%	4.0%	53%	No	No^a^
**Total from pats.**	15.5	May-14	35%	52%	47%	4.7%	57%		

a Signifies the out-links are subscriber-only.

## Scale and quality

‘So how do we know more bioactive chemistry is available from patents than papers?’ There are different data sets to approach this question but each has caveats. An upper limit is provided by the GVKBIO Online Structure Activity Relationship database (GOSTAR https://gostardb.com/gostar/). As a manually curated SAR-focused suite of databases for published and patented inhibitors against biological targets over the past 40 years, it currently includes 6.3 million chemical structures. A caveat is that a proportion of the patent activity measurements are binned rather than discrete values. A recent analysis of a 20-year slice of this data set provided some relevant statistics [17]. Firstly a total document ratio for patents: papers of 58:82 (thousand), secondly a compound ratio of 2.7:1 (million) and thirdly, an individual extracted structures per-document ratio of 12:46.

Additional data slices related to the patents: papers ratio can be made inside PubChem. The sum of all large patent-extracted sources (Table 1) is 15.4 million. The equivalent total for literature-linked compounds (via ChEMBL and PubMed/MeSH) is just over 1.0 million. The intersect (structures common to both) is ∼0.5 million. While this indicates an approximate patents: papers structure ratio of ∼15:1 there are caveats to what this represents in bioactivity terms, especially because none of the larger patent sources in PubChem currently connect structures directly to data. The intra-PubChem numbers are informative but it is necessary to ascertain selectivity to understand source complementarity [18]. Aspects of this are detailed in Table 1, along with metrics related to quality.

The dates indicate that IBM, SureChem and SCRIPDB are currently frozen. Additional date cutting indicates that ChEMBL releases are approximately tri-annual but Thomson (Reuters) Pharma submits every week. The stereo and E/Z filters are quality indicators (e.g. the highly curated source, ChEBI, scores 16%). The other manual extractions (ChEMBL and Thomson Pharma) score higher than the automated chemical named entity recognition (CNER) pipelines but, of the latter, SCRIPDB does better than IBM. Slicing the Mw distribution at 400 is a rough proxy for the length of name strings converted in CNER. Here again, as expected, manual extraction sources score higher (because they select complete structures in the first place). Causes for the low IBM score include R-group inclusions and the splitting of longer IUPAC names. Pragmatically, compared to the major benefits of being able to access them at all, the quality of open patent-extracted structures is of lesser importance (it may be for prior-art searching but this is a different issue). Reasons for this include: (a) the value lies primarily in the document > assay > result > compound > protein (D-A-R-C-P) relationships, so even if C (compound) has only a similarity match (e.g. due to an error in another C), the relevance of the connection can usually be resolved; (b) mistakes, isomeric variation and other forms of representational ‘noise’ in the original documents largely determine extraction quality *per se*; (c) different extracted isomers and tautomers can be connected (i.e. via the C-to-C match) or same connectivity relationships inside PubChem; (d) both objective quality measurements and structures-in-common between independent sources are important to assess for any large sets of structures (i.e. not just from patents) and (e) reassuring levels of extraction concordance are not only formally recorded within PubChem via substances (SIDs) from different patent sources, but also, in SureChEMBL, this extends to multiple intra-document and inter-document structure identities within the same patent family.

Uniqueness, indicated by structures in only one source, is a useful value indicator but not without caveats. The figures (Table 1) suggest that the SureChem CNER extraction has contributed the most novel patent structures, However, a proportion of these could be alternative representations of the same canonical forms in other sources (although this measurement is confounded for comparisons between ChEMBL and Thomson Pharma, because they share some of the same journal sources). The two-component count identifies ∼5% mixtures in SCRIPDB and SureChem (mostly salts) but IBM appear to have filtered these out. The next category is a crude lead-like molecular property filter. The sources converge at around 50–60% for patent structures. Thus, even if these do not have explicit assay results, they represent a large and generally synthetically accessible, potential bioactive chemical space. The last two columns in Table 1 refer to structure-to-document connectivity. Inside the CID records those with links to the USPTO website are processed from IBM and SCRIPDB by PubChem. They are also usefully IPC-indexed by which we can establish that of the 8.5 million structures assigned codes, 7.2 million are under C07 and 6.1 million C07D. Note that the SureChem records match SureChEMBL externally where a structure search connects to them to patent numbers (also IPC indexed) from the major offices. For Thomson Pharma, the ∼4.2 million external links are subscription-only but can be either to a patent and/or a paper (it would seem probable that the chemistry curation split is similar to GOSTAR that is ∼3:1 patents: papers).

## Relationship annotation in databases

**Figure 2:**
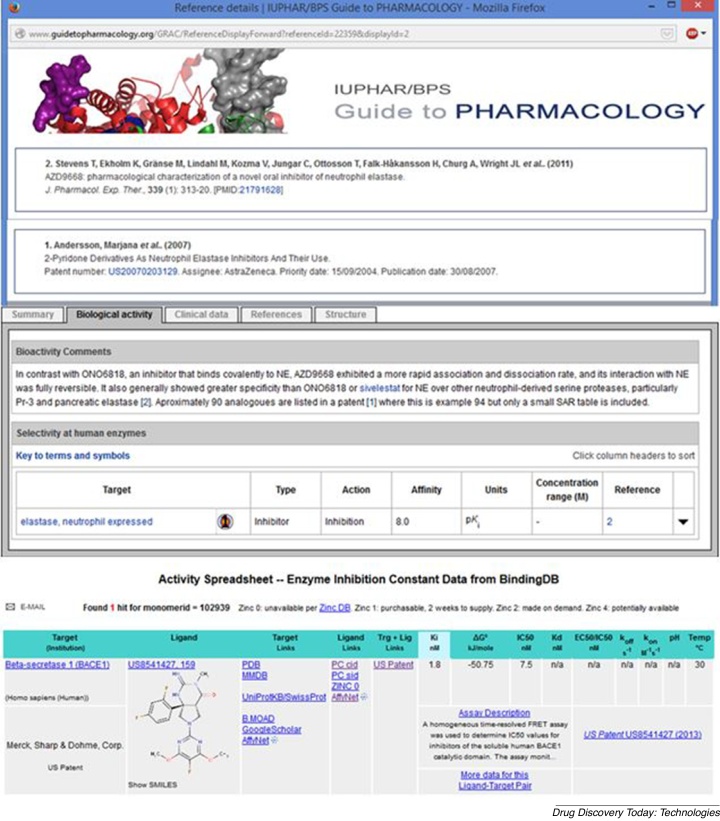
Examples of curated and annotated database mappings from patents. The upper panel shows part of the Guide to PHARMACOLOGY (GToPdb) entry (http://www.guidetopharmacology.org/GRAC/LigandDisplayForward?ligandId=6476) for AZD9668 as a clinically tested neutrophil elastase (UniProt P08246) inhibitor. The curator's notes and the two references are shown; including a SureChEMBL link to the patent US20070203129 [19] (additional connectivity for this entry has been added for the next update). The lower panel shows one of the views on BindingDB for PubChem CID 44247663 from a US8541427 [20] on BACE1 inhibitors.

The BACE1 examples above (Fig. 1) show that D-A-R-C-P mapping from an individual document requires curation. Any scaled-up availability of this (analogous to that done for papers in ChEMBL) has hitherto been a feature of a limited number of commercial databases. Nevertheless, example entries from new initiatives in two open databases are shown (Fig. 2).

In GtoPdb the paper and the patent were connected by curatorially establishing identical structures for CID 46861623 and the IPUAC name. The journal publication focuses on the pharmacology of AZD9668 (i.e. this is not an SAR paper and may therefore neither be picked up by ChEMBL nor consequently PubChem BioAssay). The complementarity of the curated pointer to the patent for anyone interested in this drug is both an extensive set of analogues and unpublished data. Note that AZD9668, as patent example 94, has Kd results converted to Ki but an IC50 only in the paper. However, seven analogues have IC50 data in the patent, including the most potent (example 32 at 3 nM) as CID 11478818 (n.b. the patent may have been filed before AZD9668 was selected for development). While a limited number of GtoPdb entries have patent connections so far, more are being added, particularly for those clinical candidates with little or no SAR in papers.

The patent curation in BindingDB, initiated in Sept 2013, is also of high utility but takes a different approach. In this case, the selection of recent US patents is protein target-based. The BACE1 filing in Fig. 2 has 42 example structures (via CWUs) manually aligned with their activity data from the patent tables but intersected with PubChem CIDs, BindingDB SIDs and a short assay description. For example, the record (http://www.bindingdb.org/data/mols/tenK10/MolStructure_102939.html), was extracted from US8541427 [20]. While this does not locate the structure within the document (i.e. searching SureChEMBL, via CID 44247663, connects to the image for example 10), it does allow the curated set to be directly retrieved as a CID list with the PubChem query ‘US8541427’. This can be done for any of the 367 BindingDB patents (Oct 2014) covering 32 670 compounds with target-mapped activity results.

## Conclusions

The options to mine patent data from individual documents up to large extracted structure sets are expanding in open resources. For example, SureChEMBL has reached 15.6 million *in situ* at 80 K novel structures per month (Dr. G. Papadatos, RDKit UGM, presentation Nov 2014). Paradoxically, patents are fully accessible for text-mining, in contrast to most of the literature. Of the patent-extracted structures already in PubChem over 9 million are within the property boundaries for potential bioactivity and ∼0.5 million intersect with identical structures from papers, via ChEMBL and/or PubMed. Future challenges will include abstracting D-A-R-C-P relationships and synergistically intersecting these with the analogous relationships and entities identified from the literature, as already demonstrated by BindingDB and GtoPdb.

## Conflict of interest

The author declares no conflict of interest.

## Note added in proof

SureChEMBL have just (Feb 2015) submited 14.6 million patent-extracted structures to PubChem. These displace the previous SureChem entries, include document links and increase the patent chemisty coverage by several million (depending on exact overlaps with other sources).
